# Evaluation of *Rumex hastatus* leaves against hepatic fibrosis: a rat model

**DOI:** 10.1186/s12906-017-1943-5

**Published:** 2017-08-30

**Authors:** Sumaira Sahreen, Muhammad Rashid Khan, Rahmat Ali Khan

**Affiliations:** 1 Botanical Sciences Division, Pakistan Museum of Natural History, Garden Avenue, Shakarparian, Islamabad, Pakistan; 2 Department of Biochemistry, Faculty of Biological Sciences, Quaid-i-Azam University Islamabad, Islamabad, Pakistan; 3 Department of Biotechnology, Faculty of Biological Sciences, University of Science and Technology, Bannu, KPK 28100 Pakistan

**Keywords:** *Rumex hastatus*, leaves, Carbon tetrachloride, Hepatotoxicity, Oxidative stress, Fibrosis

## Abstract

**Background:**

*Rumex hastatus* leaves have been widely used as food additive and for the treatment of various liver ailments. According to our previous studies, ethyle acetate (ERL) and methanolic (MRL) fractions of *R. hastatus* leaves are an accessible source of natural antioxidants. In the present research work we arranged to investigate the *R. hastatus* leaves as hepaptoprotective agent verse hepatic damages caused by CCl_4_.

**Methods:**

During this project we divided 48 rats into eight groups randomly**.** CCl_4_-induced damages were assessed through liver function markers viz.; alkaline phosphatase (ALP), alanine transaminase (ALT), aspartate transaminase (AST), γ-glutamyltransferase (γ-GT) and lactate dehydrogenase (LDH). Changes in lipid profile were checked by measuring serum total cholesterol (TC), triglycerides (Tg), high density lipoproteins (HDL) and low density lipoproteins (LDL). Antioxidant status was checked by the activities of antioxidant enzymes, DNA damages and cellular abnormalities at histo level.

**Results:**

Administration of CCl_4_ in rats caused significant increase in liver function and lipid profile indicating hepatic damages which were restored by co-administration of *R. hastatus* extracts. Cellular and DNA damages in hepatic tissues were caused by CCl_4_ which shown clear hepatic fibrosis in addition to disturb antioxidant enzyme level. Co-treatment with various fractions of *R. hastatus* leaves regulated these markers of oxidative dysfunctions.

**Conclusion:**

From the present report it was inferred that *R. hastatus* leaves have the ability to reverse CCl_4 -_ induced hepatic damages.

## Background

Liver fibrosis is an irreversible alteration in the architecture of standard body cells and tissues caused by prolonged hepatic damages, including drug and chemical toxicity. Carbon tetrachloride (CCl_4_) is an effective hepatotoxic agent under laboratory experimentations causes oxidative damages in experimental animal models. CCl_4_ react with membrane lipids and converting into trichloromethyl radical (.CCl_3_) and finally liver cells fibrosis during chronic cases [1, 2]. Multiple reports revealed that these free radicles also depleted the antioxidant enzymatic and non-enzymatic levels which are linked to hepatic injuries and fibrosis [3]. To control such damages various ways of treatment are ineffective and few in long-term process have intolerable side effects that is why recently researcher have focused on the use of effective antioxidant for assessment against hepatic fibrosis [4]. Earlier investigations revealed that non enzymatic exogenous antioxidants like sylimarin depleted the oxidation of lipids and partially improved the hepatic damages. Similarly fruits, vegetables and medicinal plants possess active bioactive constituents which showed anti-mutagenic, anti-cancer and hepatoprotective effects [5].

*Rumex hastatus* D. Don is widely distributed in Pakistan, Afghanistan and China for the treatment of lungs bleeding and skin disorders [6]. Our previous studies verified that the *R. hastatus* leaves are used as a good antioxidant source with sufficient amount of phenolics [7]. Zhang et al. [8] by referring the use in Chinese herbal system reported seven phenolic compounds from *R. hastatus* roots. Sahreen et al. [9] reported that various fractions of *Rumex hastatus* roots showed protective effects against hepato and testicular toxicity caused by CCl_4_. Singh et al. [10] reported that *Rumex hastatus* D. Don stem and roots has potent anti-nociceptive, anti-inflammatory and anti-pyretic activities in addition to prevent neurological disorders [11, 12]. On this bases in the present study we arranged to investigate the hepatoprotective effects of *R. hastatus* against CCl_4_ induced oxidative damages in rats.

## Methods

### Plant collection and extractions

*R. hastatus* was collected from District Mansehra, Pakistan, recognized and a specimen voucher was submitted in the University Herbarium for further record. Leaves were shed dried, cut into small pieces and ground. Powder sample (5 kg) was extracted into 70% (10 L) methanol and further fractioned through solvent-solvent extraction [7] and kept in refrigerator hepatoprotective investigations.

### Ethical committee recommendation for toxicity studies

Acute toxicity was checked using ARRIVE guidelines, reporting without abnormal observations fulfilling recommendation of ethical committee.

### Animals and treatment

Six-week-old 48 male Sprague-Dawley rats (180 ± 10) g were divided randomly into eight groups (6 rats of each group). Group I was control; Group (II) was vehicle control Group (III) rats received CCl_4_ (0.5 ml/kg of b.w.; 20% CCl_4_/olive oil) twice a week for eight weeks; (IV) was administered 50 mg/kg of b.w., silymarin; (V & VII) was administered 200 mg/kg of b.w., fraction of ethyle acetate dose group; (VI & VIII) the 200 mg/kg of b.w., methanol extract and groups (IV, V and VI) 0.5 ml/kg b.w., of CCl_4_ intragestrictlly twice a week for eight weeks. After the end of experiment all the rats were forfeited; prior to the removal of tissue, blood was obtained via cardiac perforation. The serum was kept at −80 °C after parting until it was examined. Half part of the liver was treated for enzyme and genotoxicity assays while the other part was proceed for cellular analysis.

### Serum markers assessment

Serum analysis of various liver marker enzymes such as ALT, AST, ALP, Level of LDH, γ-GT and lipid profile (Triglycerides, total cholesterol, LDL and LDH) were estimated by using standard AMP diagnostic kits (Stattogger Strasse 31b 8045 Graz, Austria).

### Antioxidant enzymes status

Liver tissue homogenate (10%) was arranged phosphate buffer. BSA method was used for determination of protein [13]. Activities of enzymes CAT, POD [14] and SOD were determined using standard protocol [15].

### Glutathione-S-transferase assay (GST)

Glutathione-S-transferase activity was assayed by the method of Habig et al. [16]. The reaction mixture was consist of 1.475 ml phosphate buffer (0.1 mol, pH 6.5), 0.2 ml reduced glutathione (1 mmol), 0.025 ml (CDNB) (1 mmol) and 0.3 ml of homogenate in a total volume of 2.0 ml. The changes in the absorbance were recorded at 340 nm and enzymes activity was calculated as nmol CDNB conjugate formed/min/mg protein using a molar extinction coefficient of 9.6 × 10^3^ M^−1^ cm^−1^.

### Glutathione reductase assay (GR)

Glutathione reductase activity was determined by method of Carlberg and Mannervik [17]. The reaction mixture consisted of 1.65 ml phosphate buffer: (0.1 mol; pH 7.6), 0.1 ml EDTA (0.5 mmol), 0.05 ml oxidized glutathione (1 mmol), 0.1 ml NADPH (0.1 mmol) and 0.1 ml of homogenate in a total volume of 2 ml. Enzyme activity was quantitated at 25 °C by measuring disappearance of NADPH at 340 nm and was calculated as nmol NADPH oxidized/min/mg protein using molar extinction coefficient of 6.22 × 10^3^ M^−1^ cm^−1^.

### Glutathione peroxidase assay (GSH-Px)

Glutathione peroxidase activity was assayed by the method of Mohandas et al. [18]. The reaction mixture was consist of 1.49 ml phosphate buffer (0.1 mol; pH 7.4), 0.1 ml EDTA (1 mmol), 0.1 ml sodium azide (1 mmol), 0.05 ml glutathione reductase (1 IU/ml), 0.05 ml GSH (1 mmol), 0.1 ml NADPH (0.2 mmol), 0.01 ml H_2_O_2_ (0.25 mmol) and 0.1 ml of homogenate in a total volume of 2 ml. The disappearance of NADPH at 340 nm was recorded at 25 °C. Enzyme activity was calculated as nmol NADPH oxidized/min/mg protein using molar extinction coefficient of 6.22 × 10^3^ M^−1^ cm^−1^.

### Quinone reductase assay (QR)

The activity of quinone reductase was determined by the method of Benson et al. [19]. The 3.0 ml reaction mixture consisted of 2.13 ml Tris-HCl buffer (25 mM; pH 7.4), 0.7 ml BSA, 0.1 ml FAD, 0.02 ml NADPH (0.1 mM), and 0.l ml of homogenate. The reduction of dichloro phenol indophenol (DCPIP) was recorded at 600 nm and enzyme activity was calculated as nM of DCPIP reduced/min/mg protein using molar extinction coefficient of 2.1 × 10^4^ M^−1^ cm^−1^.

### Reduced glutathione assay (GSH)

Reduced glutathione was estimated by the method of Jollow et al. [20]. 1.0 ml sample of homogenate was precipitated with 1.0 ml of (4%) sulfosalicylic acid. The samples were kept at 4 °C for 1 h and then centrifuged at 1200×g for 20 min at 4 °C. The total volume of 3.0 ml assay mixture contained 0.1 ml filtered aliquot, 2.7 ml phosphate buffer (0.1 mol; pH 7.4) and 0.2 ml DTNB (100 mmol). The yellow color developed was read immediately at 412 nm on a SmartSpecTM plus Spectrophotometer. It was expressed as μmol GSH/g tissue.

### Estimation of lipid peroxidation assay (TBARS)

The assay for lipid peroxidation was carried out by the modified method of Iqbal et al. [21]. The reaction mixture in a total volume of 1.0 ml contained 0.58 ml phosphate buffer (0.1 mol; pH 7.4), 0.2 ml homogenate sample, 0.2 ml ascorbic acid (100 mmol), and 0.02 ml ferric chloride (100 mmol). The reaction mixture was incubated at 37 °C in a shaking water bath for 1 h. The reaction was stopped by addition of 1.0 ml 10% trichloroacetic acid. Following addition of 1.0 ml 0.67% thiobarbituric acid, all the tubes were placed in boiling water bath for 20 min and then shifted to crushed ice-bath before centrifuging at 2500×g for 10 min. The amount of TBARS formed in each of the samples was assessed by measuring optical density of the supernatant at 535 nm using spectrophotometer against a reagent blank. The results were expressed as nmol TBARS/min/mg tissue at 37 °C using molar extinction coefficient of 1.56 × 10^5^ M^−1^ cm^−1^.

### DNA fragmentation assay

DNA fragmentation assay was conducted using the procedure of Wu et al. [22] with some modifications. The tissue (50 mg) was homogenized in 10 volumes of a TE solution pH 8.0 (5 mmolTris-HCl, 20 mmol EDTA) and 0.2% triton X-100. 1.0 ml aliquot of each sample was centrifuged at 27,000×g for 20 min to separate the intact chromatin (pellet, B) from the fragmented DNA (supernatant, T). The pellet and supernatant fractions were assayed for DNA content using a freshly prepared DPA (Diphenylamine) solution for reaction. Optical density was read at 620 nm through spectrophotometer (SmartSpecTMPlus Spectrophotometer catalog # 170–2525). The results were expressed as amount of fragmented DNA by the following formula;

$$ \mathrm{Fragmented}\ \mathrm{DNA}=\mathrm{Tx}100/\mathrm{T}+\mathrm{B}. $$

### DNA ladder assay

DNA was isolated by the methods of Wu et al. [22] to estimate DNA damages. 5 μg DNA of rats were separately loaded in 1.5% agarose gel containing 1.0 μg/ml ethidium bromide including DNA standards (0.5 μg per well). Electrophoresis was performed for 45 min at 100 Volt. After electrophoresis gel was studied under gel doc system and was photographed through digital camera.

### Assessment of cellular changes

Microscopic analysis was conducted to check the cellular abnormalities under light microscope (DIALUX 20 EB) at 10X magnifications.

### Statistical analysis

To determine the treatment effects, one-way analysis of variance was carried by computer software SPSS 13.0. Level of significance among the various treatments was determined by LSD at 0.05% and 0.01% level of probability.

## Results

### Effects on liver and lipid profile

Administration of CCl_4_ caused a significant (*p* < 0.05) elevation of liver function profile viz.; γ-GT, AST, ALT, LDH, ALP and lipid profile as comparatively to non-treated normal rats (Tables 1 and 2). Co-administration significantly (*p* < 0.05) reversed the level of the reported enzymes and lipid profile up to normal level. Silymarin presented same effects on erasing the toxic effects on liver function and lipids parameters viz.; total cholesterol, HDL, LDL, and total triglycerides (Tables 1 and 2).

**Table 1:** Effects of various fractions of *R. hastatus* leaves on liver function tests

Group	AST (U/l)	ALT (U/l)	ALP (U/l)	γ-GT (U/l)	LDH (U/l)
Control	98.51 ± 3.13^++^	83.93 ± 2.11^++^	153.57 ± 6.18^++^	2.23 ± 0.25^++^	49.72 ± 3.60^++^
Oil + DMSO	97.86 ± 3.74^++^	84.41 ± 2.78^++^	151.19 ± 4.29^++^	2.20 ± 0.16^++^	50.29 ± 2.31^++^
CCl_4_	213.57 ± 5.70**	219.32 ± 3.80**	450.84 ± 6.5**	5.89 ± 0.36**	103.62 ± 3.62**
Sily + CCl_4_	134.37 ± 3.42^++^	129.28 ± 2.72^++^	223.43 ± 8.83^++^	3.09 ± 0.52^++^	68.72 ± 2.13^++^
ELR + CCl_4_	141.56 ± 4.86^++^	131.13 ± 2.81^++^	263.72 ± 12.93^++^	3.56 ± 0.27^++^	71.71 ± 2.52^++^
MLR + CCl_4_	157.82 ± 3.62^++^	136.74 ± 2.89^++^	276.49 ± 11.67^++^	3.24 ± 0.71^++^	73.26 ± 2.48^++^
ELR alone	96.81 ± 3.21^++^	85.34 ± 1.52^++^	153.26 ± 8.32^++^	2.26 ± 0.16^++^	48.43 ± 1.34^++^
MLR alone	95.34 ± 2.34^++^	84.34 ± 2.34^++^	149.37 ± 7.25^++^	2.04 ± 0.10^++^	49.36 ± 2.65^++^

Values are Mean ± SD (06 number), Sily = Silymarin

**indicate significance from the control group at *P* < 0.05 and *P* < 0.01 probability level

^++^indicate significance from the CCl_4_ group at *P* < 0.05 and *P* < 0.01 probability level

**Table 2:** Effects of various fractions of *R. hastatus* leaves on lipid profile

Group	Triglycerides (mg/dl)	Total cholesterol (mg/dl)	HDL (mg/dl)	LDL (mg/dl)
Control	125.56 ± 3.16^++^	32.43 ± 1.13^++^	33.45 ± 3.78^++^	30.52 ± 1.03^++^
Oil + DMSO	124.35 ± 2.98^++^	33.00 ± 2.34^++^	32.92 ± 1.43^++^	30.98 ± 1.26^++^
CCl_4_	239.55 ± 2.99**	78.07 ± 1.12**	65.64 ± 2.53**	42.98 ± 0.37**
Sily + CCl_4_	169.75 ± 4.67^++^	46.83 ± 1.38^++^	43.98 ± 2.75^++^	34.67 ± 0.15^++^
ELR + CCl_4_	177.42 ± 4.25^++^	40.18 ± 2.28^++^	40.72 ± 2.49^++^	35.14 ± 0.11^++^
MLR + CCl_4_	187.77 ± 3.54^++^	50.34 ± 3.18^++^	46.23 ± 2.98^++^	33.56 ± 0.08^++^
ELR alone	121.12 ± 1.44^++^	30.41 ± 1.24^++^	30.30 ± 1.01^++^	29.79 ± 1.00^++^
MLR alone	122.06 ± 0.89^++^	30.86 ± 1.26^++^	28.36 ± 2.90^++^	30.29 ± 1.31^++^

Values are Mean ± SD (06 number), Sily = Silymarin

**indicate significance from the control group at *P* < 0.05 and *P* < 0.01 probability level

^++^indicate significance from the CCl_4_ group at *P* < 0.05 and *P* < 0.01 probability level

### Effects of extract on antioxidant status

Endogenous antioxidant enzymes play a crucial role in the scavenging of free radicles to improve human health. Effects of various fractions of leaves extract of *R. hastatus* on antioxidant profile are presented in Table 3. Treatment of CCl_4_ considerably (*p* < 0.05) reduced the protein contents and antioxidant enzymes activity comparatively to normal non treated rats. Co-administration of fractions revealed significant enhancement the antioxidant enzyme level near to control group versus the CCl_4_ toxicity.

**Table 3:** Effects of various fractions of *R. hastatus* leaves on tissue proteins and antioxidant enzyme

Group	Protein (μg/mg tissue)	CAT (U/min)	POD (U/min)	SOD (U/mg protein)	TBARS (nM/min/mg protein)
Control	3.45 ± 0.027^++^	6.16 ± 0.14^++^	15.48 ± 0.20^++^	5.33 ± 0.08^++^	2.46. ± 0.15^++^
Oil + DMSO	3.53 ± 0.007^++^	6.19 ± 0.30^++^	15.48 ± 0.32^++^	5.21 ± 0.41^++^	2.48 ± 0.20^++^
CCl_4_	1.26 ± 0.013**	3.02 ± 0.24**	7.54 ± 0.51**	2.06 ± 0.16**	6.83 ± 0.46**
Sily + CCl_4_	2.79 ± 0.018^++^	5.00 ± 0.46^++^	12.94 ± 0.18^++^	4.10 ± 0.52^++^	3.81 ± 0.42^++^
ELR + CCl_4_	2.53 ± 0.028^++^	5.32 ± 0.34^++^	12.65 ± 0.34^++^	3.98 ± 0.17^++^	3.90 ± 0.37^++^
MLR + CCl_4_	2.48 ± 0.041^++^	4.65 ± 0.39^++^	12.09 ± 0.48^++^	3.85 ± 0.35^++^	4.09 ± 0.42^++^
ELR alone	3.48 ± 0.008^++^	6.28 ± 0.22^++^	15.42 ± 0.17^++^	5.39 ± 0.18^++^	2.46. ± 0.07^++^
MLR alone	3.47 ± 0.029^++^	6.40 ± 0.10^++^	15.76 ± 0.10^++^	5.52 ± 0.08^++^	2.73. ± 0.49^++^

Values are Mean ± SD (06 number). Sily = Silymarin

**indicate significance from the control group at *P* < 0.05 and *P* < 0.01 probability level

^++^indicate significance from the CCl_4_ group at *P* < 0.05 and *P* < 0.01 probability level

TBARS play an important role in the assessment of antioxidant activities. Treatment of CCl_4_ caused marked (*p* < 0.05) increase in the level of TBARS which was reimbursed by administration of ethyl acetate (ELR) and methanol extract (MLR) near to control group. Non significant relationship was found by feeding fractions of *R. hastatus* leaves alone against the control group.

Table 4 revealed the effects of fractions on the activities of GR, GPx, QR, GST, GSH and damages of DNA. CCl_4_ treatment significantly reduced (*p* < 0.05) the GSH contents and activities of QR, GST GPx, and GR comparatively to non-treated normal rats. Co-administration of plant extracts and silymarin significantly (*p* < 0.05) reversed the oxidative abnormalities to normal level.

**Table 4:** Effects of various fractions of *R. hastatus* leaves on phase II antioxidant enzymes and DNA fragmentation

Group	GST (nM/min/mg protein)	GPx (nM/min/mg protein)	GR (nM/min/mg protein)	GSH (μM/g tissue)	QR (nM/min/mg protein)	DNA damages %
Control	240.51 ± 6.19^++^	180.42 ± 3.25^++^	200.29 ± 3.13^++^	32.86 ± 3.22^++^	148.55 ± 2.22^++^	8.13 ± 1.03^++^
Oil + DMSO	248.35 ± 3.75^++^	179.31 ± 3.56^++^	202.42 ± 3.16^++^	33.41 ± 4.05^++^	149.63 ± 1.79^++^	8.28 ± 2.08^++^
CCl_4_	125.85 ± 5.79**	83.48 ± 2.39**	103.38 ± 2.87**	12.36 ± 2.68**	78.65 ± 1.37**	43.68 ± 1.37**
Sily + CCl_4_	196.00 ± 5.38^++^	148.84 ± 2.77^++^	176.32 ± 2.17^++^	24.75 ± 3.72^++^	120.17 ± 1.57^++^	14.02 ± 1.80^++^
ELR + CCl_4_	180.58 ± 5.39^++^	132.23 ± 2.38^++^	170.73 ± 2.26^++^	25.27 ± 2.38^++^	115.17 ± 1.21^++^	16.31 ± 2.02^++^
MLR + CCl_4_	187.48 ± 4.03^++^	120.78 ± 3.02^++^	154.92 ± 3.96^++^	21.58 ± 1.98^++^	108.75 ± 2.08^++^	14.19 ± 1.46^++^
ELR alone	249.98 ± 3.34^++^	185.23 ± 2.24^++^	201.29 ± 4.74^++^	35.29 ± 2.24^++^	149.12 ± 1.56^++^	6.89 ± 1.31^++^
MLR alone	247.34 ± 3.27^++^	184.34 ± 2.87^++^	204.38 ± 3.28^++^	34.26 ± 2.75^++^	150.12 ± 1.10^++^	7.00 ± 1.31^++^

Values are Mean ± SD (06 number). Sily = Silymarin

**indicate significance from the control group at *P* < 0.05 and *P* < 0.01 probability level

^++^indicate significance from the CCl_4_ group at *P* < 0.05 and *P* < 0.01 probability level

### Effects on DNA damages

**Fig. 1:**
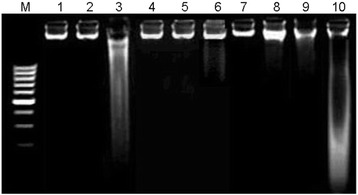
Agarose gel showing DNA damage by CCl_4_ and protective effect of *R. hastatus* leaves

DNA combine with free radicals produced by CCl_4_ making adduct and prompts DNA aberrations in rats liver tissues. Various fraction of plant extracts especially MLR and ELR as well as silymarin improved the damages comparatively to control rats as shown in Fig. 1.

### Effects of *R. hastatus* leaves on hepatic histology

**Fig. 2:**
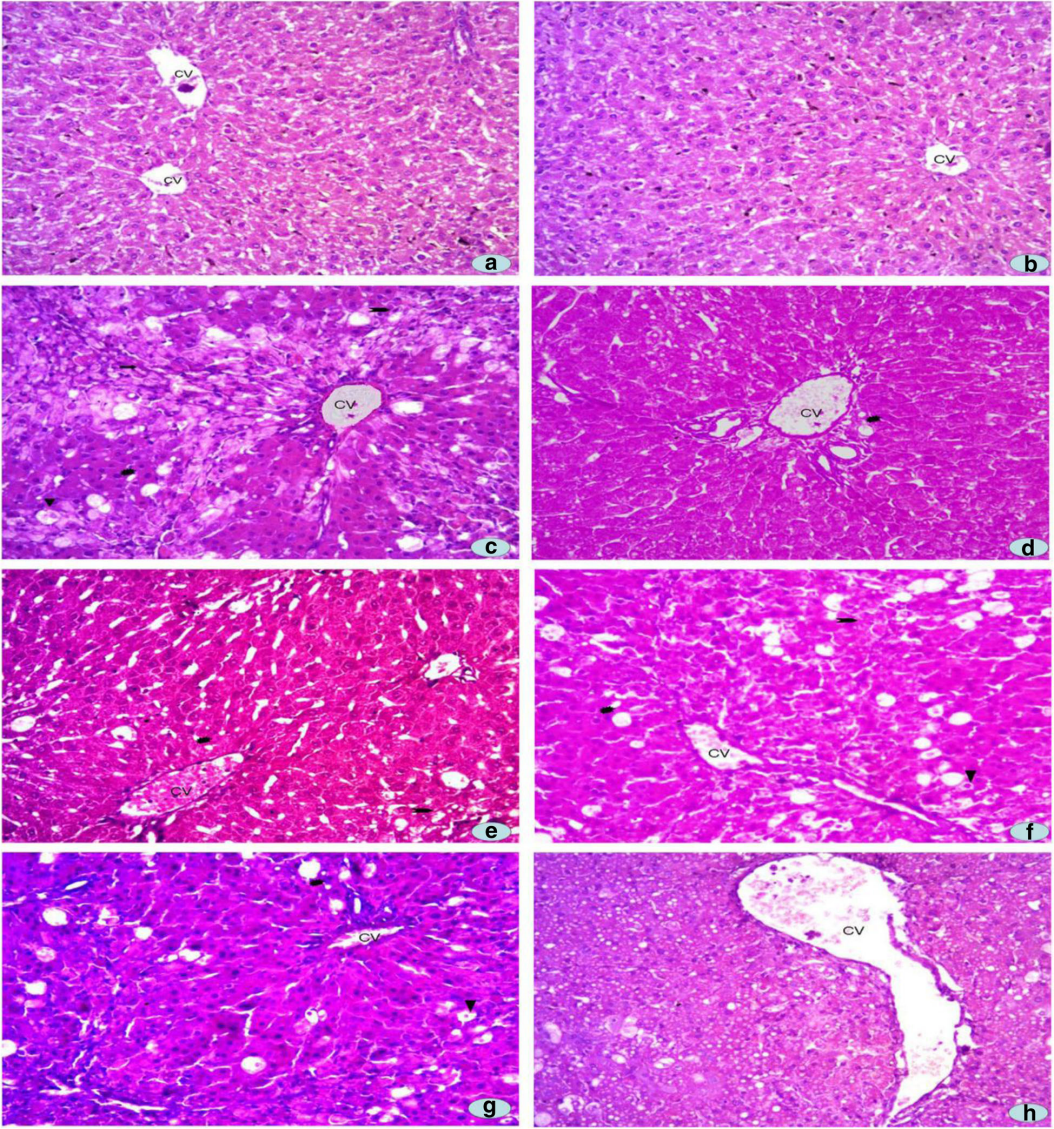
Microphotograph of rat liver (H & E stain) (→) fibrosis, (♦) cellular hypertrophy, (▲) fatty changes and ballooning: (**a**) Representative section of liver from the control group showing normal histology, (**b**) DMSO + Olive oil group, (**c**) CCl4 group, (**d**) Silymarin + CCl4 group, (**e**) ELR + CCl4 group, (**f**) MLR + CCl4 group, (**g**) ELR group, (**h**) MLR group

Cellular changes provide a strong support for biochemical and molecular reports. H & E staining were used for determination of the cellular observations as shown in Fig. 2. CCl_4_ administration produced noticeable amplification in cellular hypertrophy, inflammatory cells infiltrations, fatty changes, ballooning, the formation of septa, and degeneration of the lobular shape, necrotic foci, congestion and dilation of blood central vein. Co-administration especially ELR and MLR lessened the hepatic damages. Comparable comments were noted by the action of silymarin provides a best result for hepatic biochemical changes.

## Discussion

Phenolic and poly phenolic compounds lay a crucial role in detoxification of oxidative stress which was present in sufficient quantity in *R. hastatus* leaves [7]. Results of current in vivo project confirmed the protecting probable of *R. hastatus* leaves by reversing the CCl_4_ induced hepatic damages. Rats have been used as trial animal models to assess the hepatic degenerations caused by CCl_4_. The toxic effects observed by this induction are same as that of hepatitis. Induction of CCl_4_ caused generation of trichloromethyl free radical (CCl_3_) in the presence of P_450_ which further triages lipid peroxidation and hepatic injuries [23]. Lipid peroxidation causes change in the properties of biological molecules and pathogenesis of various diseases.

It has been reported that lipid peroxidation induced genomic overexpression of fibrogenic cytokines and proliferation of collagen which further activates the hepatic stellate cells [4]. Significant results were reported about plant extract and its fractions in amelioration of the CCl_4_ induced injuries [24]. Kupffer cells generating free radicals which causes increase in serum marker enzymes and structural changes in hepatic cells [25]. In our current research co-administration of fractions significantly reduced the serum marker enzymes and proving to improve the damages of structural integrity of the hepatocellular membrane. Similar finding were reported with other study showing anti-fibrotic effects [26].

Fibrosis of liver is a critical community health issue. Liver shows a central role in maintaining cholesterol level. Our results revealed that CCl_4_-induced hepatic fibrosis and disturbed the total cholesterol (TC) triglycerides, LDL, and HDL levels which are associated with heart disease. Co-administration of *R. hastatus* leaves extracts markedly reverses the toxic effects. Similar reports were obtained while working on Noni fruit juice against CCl_4_ induced chronic liver damage in female SD rats [5].

Antioxidant enzymes are answerable for catalytic dismutation of highly reactive lethal activists [27]. In our current study various fractions of *R. hastatus* leaves considerably controlled the liver antioxidant enzymes like CAT, POD and SOD representing the failure in oxidative injury produced by CCl_4_.

Glutathione scheme comprises GSH, GPx, and GST. GSH is a non-enzymatic antioxidant contents taking part in improving and balancing of antioxidant system via detoxification of free radical [28] as well as coordinates other phase II antioxidant enzymes [29, 30]. Our results show significant reduction in the activity of phase II metabolizing enzymes by the induction of CCl_4_ in rats which prove the free radical induced hepatic damages. Co-administration of *R. hastatus* leaves extracts reduced the CCl_4_ toxicity, by elevation of GR, GST, quinone reductase and GPx activities comparatively. Other study of co-administration of *Coriandrum sativum* in rats against CCl_4_ induced hepatotoxicity [30]. The lipid peroxidation (LPO) is an autocatalytic progression combine with hepatic membrane causes hepatotoxicity which finally induces cell death, aging and cancer [31, 32]. In our current research CCl_4_ administration caused marked increase in hepatic MDA level representing amplified oxidation of lipid which was restored by co-administration various fractions of *R. hastatus* leaves and confirmed to be a potent hepatprotective agent.

It has been examined that peroxidation of lipid contents by induction of CCl_4_ effect DNA structure integrity. MDA associated with DNA forming an adduct MIG (The mutagenic pirimedo-purinone adduct of deoxy-guanosine) by producing reactive species [19, 33]. Our current reports shows that CCl_4_ induction caused significant DNA damages quantitatively as well as quantitatively comparatively to non-treated rats. Various fractions considerably condensed the % DNA damages which were also shown by banding pattern. Same reports are obtained by other studies [21].

Cellular architecture provides a direct indication for assessment the protective effect of various fractions of leaves extract (*R. hastatus*) which provides a clear indication with support of biochemical markers. Intense changes in the LFTs intensely represent the histological indication of cellular degenerations [33, 34]. CCl_4_ administration caused broad infiltration of lymphocyte, massive fatty changes, cellular hypertrophy, gross necrosis, and collagen deposition which was distinctly reduced by co-treatment of various fractions as well as silymarin. Our results revealed similar exploration which is covenant with previous report [35] during evaluation of extracts against hepatic injuries caused in rats.

## Conclusion

Results of the current study revealed that *R. hastatus* leaves are strong antioxidant and capable to keep the liver from CCl_4_-induced liver fibrosis and provide some automatous indication for why indigenous people of Southeast Asia found it useful for treating liver ailments as well as food additive.
